# Physical activity measured with wrist and ankle accelerometers: Age, gender, and BMI effects

**DOI:** 10.1371/journal.pone.0195996

**Published:** 2018-04-27

**Authors:** Veronica Ramirez, Ehsan Shokri-Kojori, Elizabeth A. Cabrera, Corinde E. Wiers, Kathleen Merikangas, Dardo Tomasi, Gene-Jack Wang, Nora D. Volkow

**Affiliations:** 1 National Institute on Alcohol Abuse and Alcoholism, Bethesda, Maryland, United States of America; 2 National Institute of Mental Health, Bethesda, Maryland, United States of America; 3 National Institute on Drug Abuse, Bethesda, Maryland, United States of America; Vanderbilt University, UNITED STATES

## Abstract

Physical activity (PA) is associated with various aspects of physical and mental health and varies by age and BMI. We aimed to compare PA measures obtained with wrist and ankle accelerometers and characterize their associations with age and BMI. We assessed PA mean and PA variability (indexed by coefficient of variation (CV)) at daytime and nighttime periods for seven consecutive days (*M* = 152.90 h) in 47 healthy participants (18–73 years old, 21 females). Diurnally, mean PA for both ankle and wrist and CV of PA for ankle decreased from the first to the second half of daytime (*p* < 0.05). There were no differences in mean PA between wrist and ankle at any time-period (*p* > 0.2). CV of ankle PA at daytime was significantly higher than wrist PA (*p* < .0001). The opposite pattern was observed at nighttime (*p* < .0001). Pearson correlation analyses were performed to assess the associations between wrist (or ankle) PA and age and BMI. Mean daytime (but not nighttime) activity for wrist and ankle decreased significantly with age (*p* < .05). PA variability also decreased with age for wrist and ankle during daytime and for ankle during nighttime (*p* < .05). BMI was negatively associated with wrist daytime PA variability (*p* < .05). There were no gender effects on activity measures. These findings indicate that wrist and ankle mean PA measures were not significantly different but were significantly different (*p* < 0.5) for PA variability in both daytime and nighttime. Age-related decreases of PA-mean and variability were observed during daytime in wrist and ankle, whereas higher wrist daytime variability was inversely associated with BMI. These findings provide new insights into PA features in free-living environment, which are relevant for public health and may have implications for clinical assessment of neurodegenerative disorders impacting PA and their interaction with demographics.

## Introduction

Physical activity (PA) is associated with a wide range of health outcomes, demographics, developmental stages, and psychological measures [1, 2]. Specific patterns of sedentary behavior have been shown to be indicators of neurological and mental diseases [3, 4]. Regular participation in vigorous PA (mean activity), can mitigate age-related movement deficits [5, 6], reduce risk of obesity [2, 4, 7] and is negatively associated with body mass index (BMI) [8]. There is also evidence that variability in activity patterns is also important in characterizing neurological health. For example, it has been shown that in Parkinson’s disease, there is increased gait variability and walking disturbances [9]. While this is evidence that both mean and variability of PA are informative for characterizing physical and neurological health, these indices have not been well studied for different body extremities during sleep and wake cycles and within daytime periods.

Circadian rhythms influence PA-related biological processes [10] and behavioral factors [11] and research has started to recognize the importance of sleep-awake cycles when exploring gender-related [12], obesity-related [13] and age-related [14] differences of PA. Irregular patterns of sleep-wake cycles are associated with poor mental health [15], lower PA trends [16], and higher engagement in occupational than in leisure PA [17]. Scarce consideration has been given to how PA measures of upper and lower extremities between day and night periods could vary by gender, BMI, and age in healthy individuals, despite the evidence that these demographics factors impact PA mean levels [18] and PA variability [17]. In addition to sleep-wake cyclic rhythms [10], circadian changes in neurobiological systems [19] also influence patterns of activity and PA performance [20], highlighting the importance of studying effects of diurnal patterns of PA.

It has been suggested that lower extremities’ movement variability and asymmetry of steps length underlie poor balance [21], which is significantly lower in older adults than in younger people [22], indicating that PA variability of the extremities changes with age. Overall, older individuals show reduced PA [23, 24], which reflects among other factors, changes in lifestyle, decreases in skeletal muscle mass [25], flexibility, [26] and bone density [27]. These physiological changes have a higher impact on females’ functionality than in males [28, 29] which could explain the higher levels of PA engagement found in males than in females across people’s lifespan [30, 31]. Other age-related changes include increases in BMI and in visceral body fat [32], which can decrease strength, endurance, and performance [33]. More importantly, BMI levels [25] are strongly correlated with these physiological changes, while vigorous PA [34] can mitigate their effects. Nevertheless, the distinct associations between wrist and ankle PA features and age, gender, and BMI remain unclear.

Accelerometers placed on the extremities or torso objectively and unobtrusively characterize PA by measuring body acceleration in time [8, 35–39] and are increasingly being used in naturalistic settings [40, 41]. The location of actigraph monitors can provide insights into how each body part distinctively contributes to overall movement and its variability. For instance, wrist-worn PA monitors yield significantly higher variability than hip-worn monitors [42]. It is expected that within-subject changes in daily activities performed in free-setting environments result in differences in the mean and variability of activity between the upper and lower limbs. Conversely, high PA variability during daytime is distinctively present among younger populations [43], which might reflect the nature of their daily activities [23]. Similarly, adults’ PA variability is influenced by the daily structure of their activities [44], which could differently affect variability of arms [45] or ankle movements [46]. For instance, repetitive arm motions during occupational tasks [45], driving commutes [47], and mobile device use [48] would be expected to affect accelerometers’ register of activity at the wrist but not the ankle, which would remain stationary during these types of activities.

Here, we characterized differences of PA mean and variability in healthy individuals between daytime and nighttime periods for lower and upper extremities. We measured PA continuously for seven days using accelerometers positioned on wrist and ankle. The choice of wrist and ankle located monitors was due to evidence showing that PA in body extremities are differently affected by variables such as age and BMI [49], and that PA measurements of different body extremities is important for characterizing sleep-awake cycles [50]. Furthermore, we compared mean and variability of PA for the first half (P1) and the second half (P2) of daytime period to account for potential effects of circadian rhythms on PA. We studied how PA mean and variability in wrist and ankle at different time periods differ as a function of gender, age, and BMI. We predicted that males would show higher PA levels than females [31]. We predicted that age and BMI would be negatively associated with PA mean and variability, and that PA variability would differ between sleep and wake cycles as daily activities, environmental factors, and neurobiological systems may differently contribute to movements in the 24-hour cycle.

## Methods

### Participants

Sixty-two healthy volunteers were recruited through the Clinical Research Volunteer Program at National Institutes of Health to participate on a neuroimaging protocol. Fifty-four participants received the accelerometers. Two participants did not wear the accelerometers at both day and night as instructed, and accelerometer data for two participants could not be extracted due to technical issues. Finally, three subjects were excluded as outliers as their PA mean and CV for wrist or ankle (for both daytime and nighttime periods) was two standard deviations above the sample average. Thus, accelerometer data for a final sample of 47 participants (21 females and 26 males) was analyzed. The age range for this sample was 18 to 73 years old (*M =* 40.77, *SD =* 14.91) and BMI varied from 17.6 to 40.5 kg/m^2^ (*M* = 27.06, *SD* = 4.78) (See Table 1).

**Table 1:** Demographics.

	Male	Female	Total	Total Percentage (%)	Mean	SD
**Age**						
18–29	6	6	12	25.53%	23.27	3.28
30–49	14	7	21	44.68%	38.45	5.96
50–64	5	6	11	23.40%	55.98	4.06
65+	2	1	3	6.38%	72.11	1.83
**BMI**						
18.5- (underweight)	1	1	2	4.26%	17.95	0.49
18.5–24.9 (normal)	9	7	16	34.04%	23.23	1.52
25–29.9 (overweight)	14	4	18	38.30%	27.15	1.47
30+ (obese)	4	7	11	23.40%	33.77	2.99
**Ethnicity**						
Asian	2	0	2	4.26%	-	-
Black/African-American	10	15	25	53.19%	-	-
Hispanic	0	1	1	2.13%	-	-
Mixed Race	0	1	1	2.13%	-	-
White	14	4	18	38.30%	-	-
**Education**						
High School or Less	4	5	9	19.50%	-	-
Some College	3	5	8	17.02%	-	-
College +	19	11	30	63.83%	-	-
			47	100%	-	-
**Annual Income**						
<30,000	11	7	18	38.30%	-	-
30,000–74,999	10	7	17	36.17%	-	-
75,000 +	7	5	12	25.53%	-	-

Inclusion criterion included older than 18 years of age and the ability to provide written informed consent, approved by the institutional review board at NIH. Exclusion criterion for this subtask, as part of the main neuroimaging study, included past or current DSM-IV or DSM-5 diagnosis of a psychiatric disorder (including substance use disorder), history of head trauma with loss of consciousness for more than 30 minutes, use of psychoactive substances two weeks prior to the study, use of medications that could affect brain function four weeks prior to the study, body weight over 250 kg, any medical problem that would affect brain function, cardiovascular disorders, any clinically significant laboratory finding during screening process, a positive test for drugs and/or pregnancy (for females) at the time of the study. All participants underwent physical examination by a nurse practitioner to ensure there were no physical disabilities, mobility restrictions, or inability to comfortably lie flat on their back for extended periods of time. All participants were provided monetary compensation upon completing the task.

### GENEActiv accelerometer

We used GENEActiv triaxial accelerometers (Version 1.1; Activinsights Ltd., Cambridgeshire, UK) to record limbs’ acceleration along 3 orthogonal axes. All participants were asked to wear two accelerometer monitors 24 hours continuously for 7 days (sampling rate: f = 100 Hz) and to continue with their regular activities. Consistent with previous studies’ methodology [51, 52], one monitor was placed on the non-dominant wrist and one on the corresponding ankle to more closely capture overall body movements. Participants were asked not to remove the devices at any time, except during swimming activities to prevent device damage. Using GENEActiv PC Software 3.0, acceleration data were summarized within each 60-second epoch using gravity-subtracted sum of vector magnitudes of acceleration (x, y, z): ${SVM}_{S}^{g}=\sum_{t=1}^{n}((\sqrt{x_{t}^{2}+y_{t}^{2}+z_{t}^{2}}-1g)$ representing PA for each 60-second epoch [53] where, *n* (the number of measurements in the sum) is the sampling frequency times the epoch length in seconds (100 Hz × 60 s), resulting in a total of 6000 samples for every 1-minute bin (note that the acceleration values are squared in the formula regardless of their sign). Thus, the resulting SVMs for the 1-min bins were divided by 600, so that the resulting numbers have units of 0.1 *g* (i.e., *m/s*^*2*^). Using MATLAB (MathWorks, Natick, MA) software (MATLAB and Statistics Toolbox Release 2012b), 10-min sliding windows with 50% overlap were used to calculate PA’s mean and coefficient of variation (CV = standard deviation/mean) within each window. Our choice of 1-min bins is consistent with prior literature [54]. Moreover, the 10-min window length with 50% overlap (12 total windows per hour) was adequate to capture individual sleep stages and their transitions as measured previously (approximately 12 sleep events per hour) [55].

**Fig 1:**
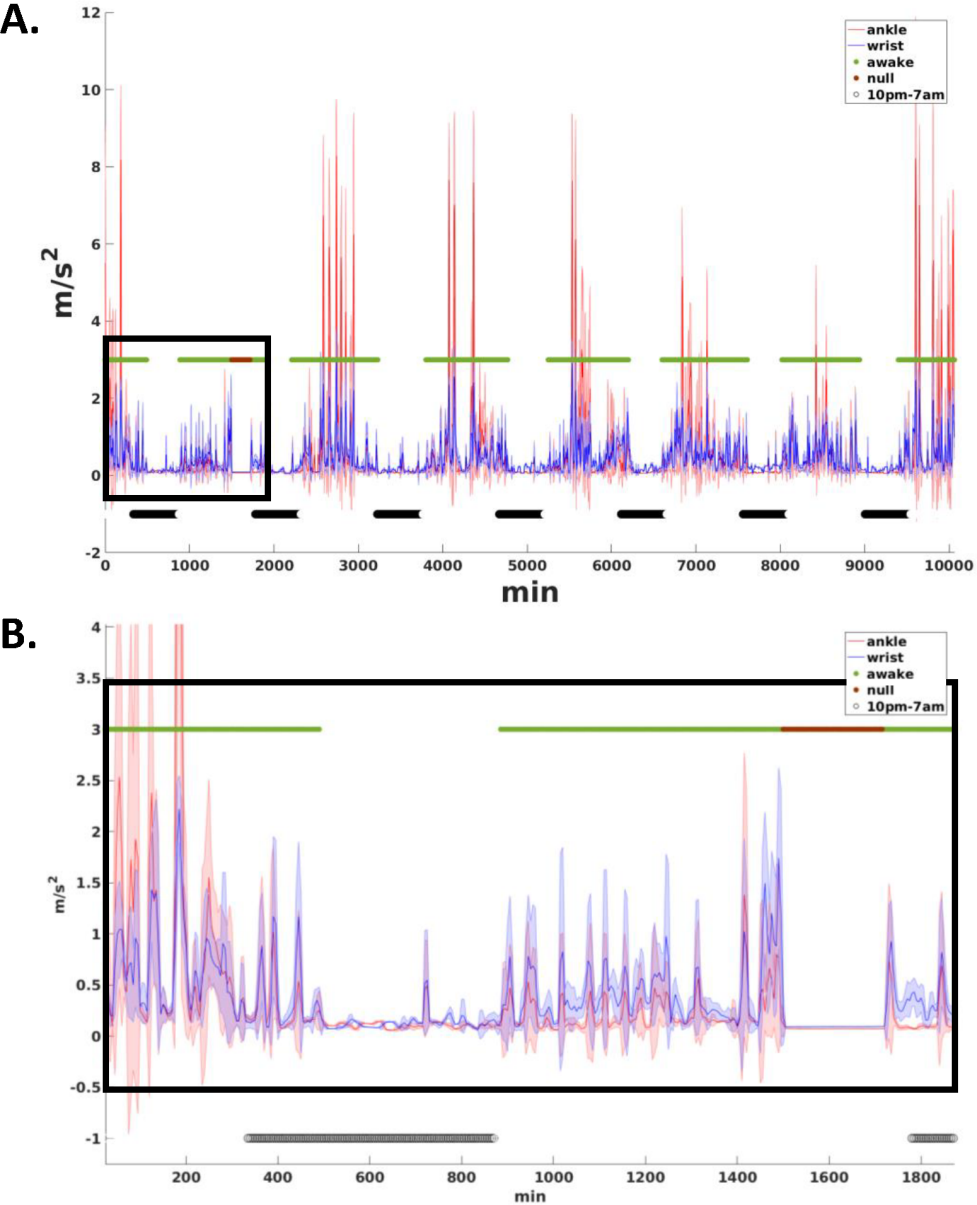
Activity count graph for a single subject for 7 consecutive days. Data was sampled into 1-min bins and averaged over 10-min windows with 50% (5 min) overlap. The solid blue line connects the ankle’s mean activity across 10-min overlapping windows, and the shaded blue area shows the standard deviation (SD) added and subtracted from the mean. The solid red line connects wrist’s mean activity across 10-min overlapping windows, and the shaded red area shows the standard deviation (SD) added and subtracted from the mean. High and low in the green line indicates the daytime and nighttime periods. The low values in the black line indicate the excluded areas associated with no-wear time or unknown activity.

Real time of activity was calculated by the sum of minutes from start time of monitor wear. The average PA mean and CV across all windows that corresponded to daytime (i.e., awake cycles) were calculated individually and guided by time: we manually identified for each day increased activity for both wrist and ankle (i.e., > 0.33 *m/s*^*2*^) during morning hours that continued for consecutive hours (Fig 1); this was classified as the beginning of each daytime period and was used to estimate PA mean and CV of daytime. The average PA mean and CV across all windows that corresponded to nighttime (i.e., sleep cycles) were calculated in the same way: we manually identified for each day considerably decreased activity for both wrist and ankle (i.e., < 0.33 *m/s*^*2*^) at evening hours that continued for several consecutive hours (Fig 1); this was classified as the beginning of each nighttime period to estimate PA mean and CV of nighttime. To capture only sleep related movements, irregular (unknown) patterns of nightly activity were also excluded from the analyses. For example, high activity counts (> 0.33 *m/s*^*2*^) for longer than 20 min continuously during nighttime periods were excluded from the data. To characterize the impact of first and second halves of daytime on PA, we segmented the daytime period into two equal parts (for each day), and mean and CV of PA for wrist and ankle for the first half (P1) and for the second half (P2) were calculated separately.

Non-wear time was manually excluded from the analysis when either wrist or ankle showed no activity (i.e., time points with activity below 0.08 *m/s*^*2*^ for over 30 min were excluded (Fig 1). We further assessed participants’ compliance by manually inspecting all the data. We found that ten participants did not wear monitors continuously for seven days, ranging from 1 to 4 missing periods. The reminder of their data was still used in our analyses. After the removal of outliers’ PA data, non-wear time, and irregular PA patterns, participants’ average number of minutes monitor wear was *M* = 152.90h (*SD* = 18.05h).

### Statistical analysis

To assess mean PA and variability of participants at sleep and wake cycles, we estimated mean and CV measures for ankle and for wrist for all time periods (i.e., daytime, nighttime, P1, and P2). Paired *t-*tests were used to compare mean and CV of PA between wrist and ankle at different time periods. Additionally, Bland-Altman agreement analysis was performed to assess bias in measuring PA due to location of measurements between wrist and ankle. Paired *t*-tests, were used to assess within-ankle and within-wrist differences of PA mean and CV for P1 and P2. Pearson product-moment correlation analyses were performed to assess the associations between wrist (or ankle) PA, and age and BMI at daytime and nighttime. Two-sample *t*-tests were used to assess the effects of gender on age, BMI and PA measures. All statistical tests were performed against a two-tailed alternative and considered significant at *p* < 0.05.

## Results

### Mean PA of wrist and ankle

**Fig 2:**
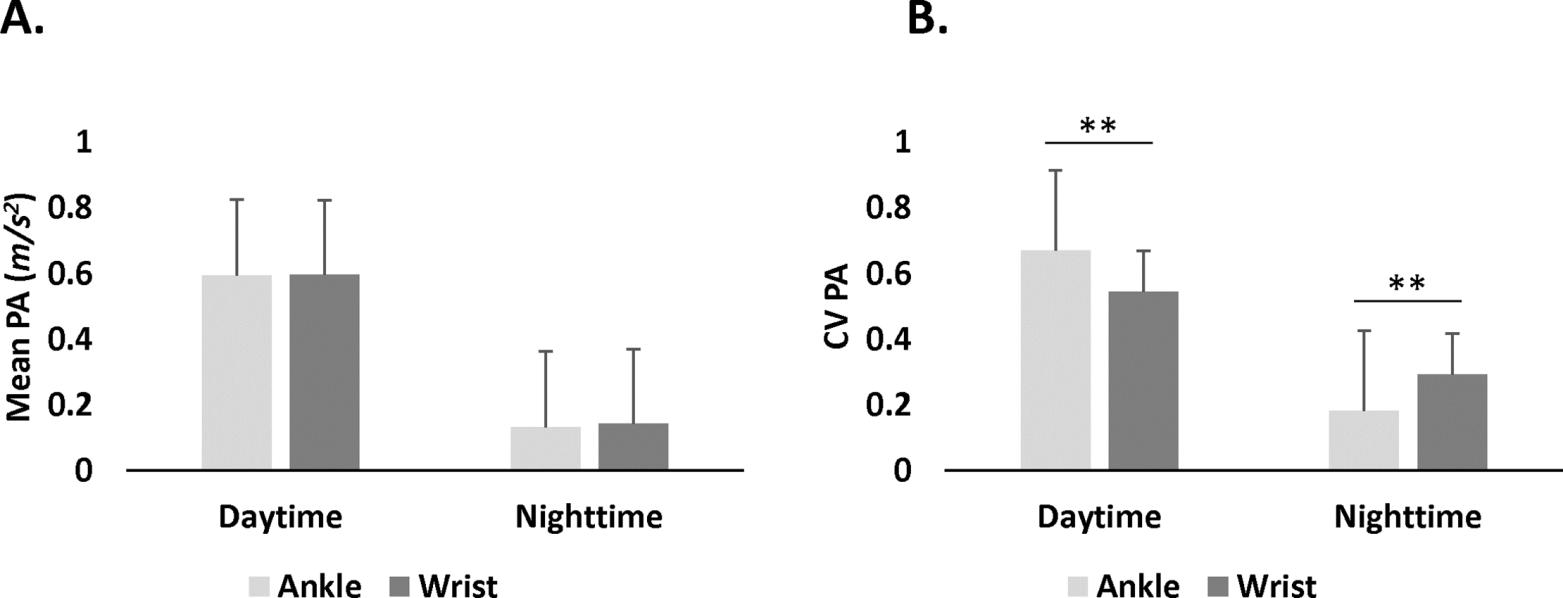
Physical activity (PA) measures for ankle and wrist at daytime and nighttime. A. Mean PA did not differ between wrist and ankle in any period. B. Coefficient of variation (CV) was different for ankle and wrist during daytime and nighttime periods. **p < .001.

Paired *t-*tests showed no difference in daytime between mean PA of wrist (*M* = 0.60 *m/s*^*2*^, *SD* = 0.14 *m/s*^*2*^) and ankle (*M* = 0.59 *m/s*^*2*^, *SD* = 0.19 *m/s*^*2*^): *t*(46) = 0.09, *p =* .93; nor in the nighttime between mean PA of wrist (*M* = 0.14 *m/s*^*2*^, *SD* = 0.04 *m/s*^*2*^) and ankle (*M* = 0.13 *m/s*^*2*^, *SD* = 0.06 *m/s*^*2*^): *t*(46) = 1.15, *p* = .26 (Fig 2A). The ankle consistently showed more inter-subject PA variability than wrist at daytime and nighttime.

### Intra-subject PA variability of wrist and ankle (CV)

Paired *t*-tests showed that intra-subject PA variability was different between wrist and ankle contingent upon period. Specifically, in daytime, ankle CV was higher (*M* = 0.67, *SD* = 0.09) than wrist CV (*M* = 0.54, *SD* = 0.06): *t*(46) = 8.99, *p* < .0001, whereas at nighttime ankle CV (*M* = 0.18, *SD* = 0.06) was lower than wrist CV (*M* = 0.29, *SD* = 0.08): *t*(46) = 11.44, *p* < .0001 (Fig 2B).

### Inter-subject correlations between ankle and wrist at daytime and nighttime

**Fig 3:**
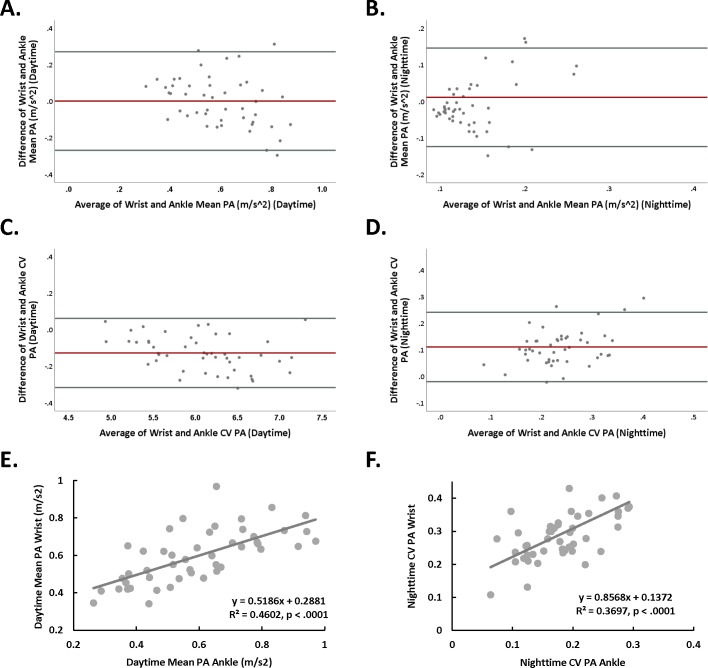
**(**A) Bland-Altman analysis for daytime mean PA for wrist and ankle. The difference between wrist and ankle mean PA plotted against the average of wrist and ankle mean PA for each subject. The redline shows a bias of 0.002 with upper and lower confidence intervals of 0.27 and -0.27 (gray lines), respectively. The equality line (y = 0) fell within the confidence intervals of average of differences (-0.04, 0.04) indicting an agreement (within-subjects) between wrist and ankle for PA mean at daytime. (B) Bland-Altman analysis for nighttime mean PA for wrist and ankle. The difference between wrist and ankle mean PA plotted against the average of wrist and ankle PA mean for each subject. The redline shows a bias of 0.012 with upper and lower confidence intervals of 0.15 and -0.12 (gray lines), respectively. The equality line (y = 0) fell within the confidence intervals of average of differences (-0.01, 0.03), indicting an agreement (within-subjects) between wrist and ankle for PA mean at nighttime. (C) Bland-Altman analysis for daytime PA CV for wrist and ankle. The difference between wrist and ankle PA CV plotted against the average of wrist and ankle PA CV for each subject. The redline shows a bias of -0.127 with upper and lower confidence intervals of 0.06 and -0.32 (gray lines), respectively. The equality line (y = 0) did not fall within the confidence intervals of average of differences (-0.15, -0.10) indicting a non-significant agreement (within-subjects) between wrist and ankle for PA CV at nighttime. This was expected due to significant differences in the PA CV between wrist and ankle at nighttime (see Fig 2B). (D) Bland-Altman analysis for nighttime PA CV for wrist and ankle. The difference between wrist and ankle PA CV plotted against the average of wrist and ankle PA CV for each subject. The redline shows a bias of 0.111 with upper and lower confidence intervals of 0.24 and -0.02 (gray lines), respectively. The equality line (y = 0) did not fall within the confidence intervals of average of differences (0.09, 0.13) indicting a non-significant agreement (within-subjects) between wrist and ankle for PA CV at nighttime. This was expected due to significant differences in the PA CV between wrist and ankle at nighttime (see Fig 2B). (E) Scatter plot of daytime mean PA of ankle plotted against the mean PA of wrist (*p* < .0001). (F) Scatter plot of nighttime PA CV of ankle plotted against the nighttime PA CV of wrist (*p <* .0001).

Correlation and Bland-Altman analyses were conducted between PA measures in daytime and nighttime (Table 2, Fig 3A–3D). We found significant correlations between wrist and ankle PA measures, specifically, mean PA of wrist and ankle were significantly correlated at daytime *r(*45) = .68, *p <* .000 (Fig 3A), but not at nighttime *r(*45) = .15, *p =* .32 (Fig 3B). In contrast, CV of wrist and ankle were not correlated at daytime *r(*45) = .21, *p =* .16 (Fig 3C) but were significantly correlated at nighttime *r(*45) = .61, *p* < .0001 (Fig 3D). In addition, scatter plots for the significant wrist and ankle associations are shown (Fig 3E and 3F).

**Table 2:** Correlations between all PA measures.

		Ankle-Day		Ankle-Night		Wrist-Day		Wrist-Night	
		Mean	CV	Mean	CV	Mean	CV	Mean	CV
**Ankle—Day**	**Mean**	1	0.51**	-0.14	0.28	0.68**	0.21	0.15	0.07
	**CV**	-	1	0.58**	0.32*	0.37*	0.21	0.21	-0.03
**Ankle—Night**	**Mean**	-	-	1	-0.25	-0.03	-0.08	0.15	-0.004
	**CV**	-	-	-	1	0.32*	0.31*	0.10	0.61**
**Wrist—Day**	**Mean**	-	-	-	-	1	0.34*	0.31*	0.32*
	**CV**	-	-	-	-	-	1	-0.34*	0.53**
**Wrist—Night**	**Mean**	-	-	-	-	-	-	1	-0.19
	**CV**	-	-	-	-	-	-	-	1

Note

* *p <* .05

** *p* < .0001.

### Age and BMI association with wrist and ankle PA

**Fig 4:**
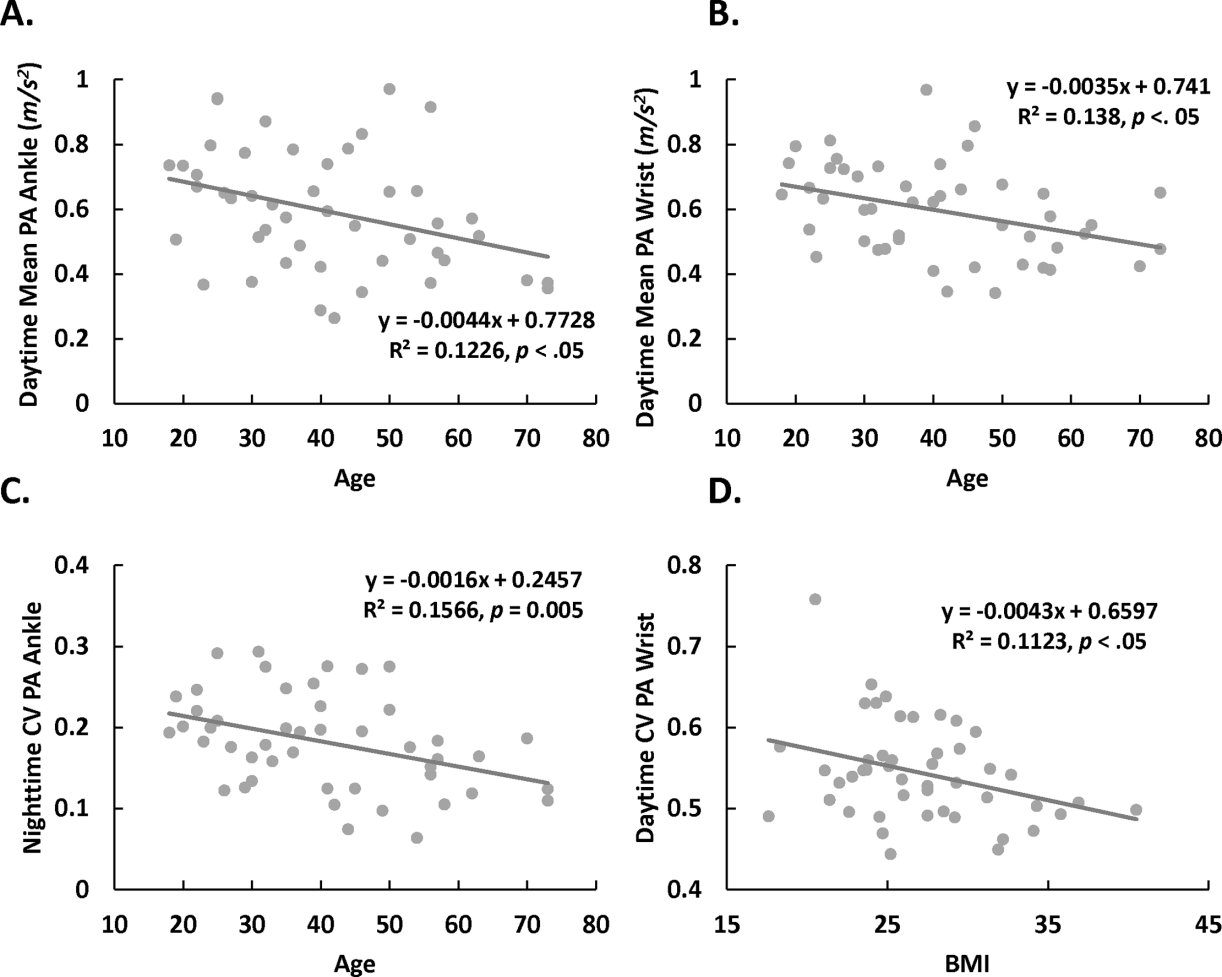
Association of physical activity (PA) measures with age and with body mass index (BMI). (A–C) Scatter plots of PA measures versus age (*p* < .02). D. Scatter plot of wrist coefficient of variation (CV) versus BMI (*p* = .021) at daytime. However, after removing one potential outlier with high CV of 0.76, this association was marginally significant (*p* = 0.06).

Age and mean PA showed a negative association for daytime, both for ankle *r*(45) = -.35, *p* = .02 (Table 3 and Fig 4A), and wrist *r*(45) = -0.37, *p* = .01 (Table 3 and Fig 4B), but nighttime correlations were not significant. Age and CV PA showed a negative correlation during daytime for ankle *r*(45) = -.31, *p* = .03 and for wrist *r*(45) = -.33, *p* = .02 (Table 3), but only for ankle during nighttime *r*(45) = -.40, *p* = .005 (Table 3 and Fig 4C).

**Table 3:** Correlations between PA measures and age and BMI.

	Ankle—Day		Ankle–Night		Wrist—Day		Wrist—Night	
	Mean	CV	Mean	CV	Mean	CV	Mean	CV
**Age**	-0.35*	-0.31*	-0.17	-0.40*	-0.37*	-0.33*	-0.19	-0.21
**BMI**	-0.07	-0.01	-0.19	0.13	-0.15	-0.34*	-0.14	-0.03

Note

*p < .05

The only significant correlation with BMI was for wrist CV during daytime *r*(45) = -.34, *p* = .021 (Table 3 and Fig 4D). However, this correlation was mostly driven by one individual, and the significance was only marginal after its removal. For our sample BMI and age were not correlated *r*(45) = .17, *p* = .25.

### Gender and PA measures of wrist and ankle

Two-sample t-tests revealed no significant differences in BMI (*p* = .851) and age (*p* = .983) between males and females. In addition, there were no gender effects of PA mean and CV for wrist or ankle at any period, *p >* 0.1 (See Table 4).

**Table 4:** PA measures by gender.

		Males	Females	*T* value	*P* value
**Ankle Daytime**	**Mean, SD**	0.06	0.06	0.1	0.923
	**CV**	0.65	0.69	-1.47	0.148
**Ankle Nighttime**	**Mean**	0.01	0.01	0.134	0.894
	**CV**	0.18	0.18	-0.315	0.754
**Wrist Daytime**	**Mean**	0.06	0.06	-0.547	0.587
	**CV**	0.55	0.54	-0.515	0.609
**Wrist Nighttime**	**Mean**	0.01	0.02	-0.999	0.323
	**CV**	0.30	0.28	1.042	0.303

Note: Mean PA values are in 0.1 *g units (i*.*e*., *m/s*^*2*^*)*

### PA of wrist and ankle within daytime segments

**Fig 5:**
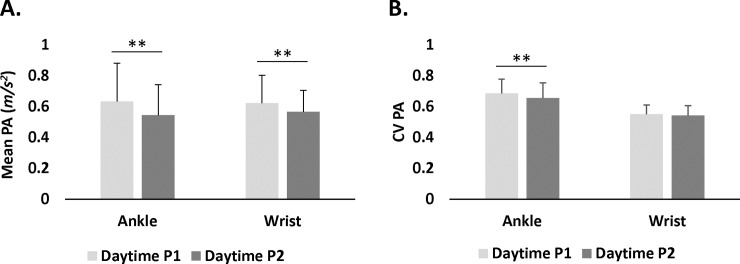
Physical activity (PA) measures for ankle and wrist for daytime P1 and daytime P2 for mean PA (A) and for CV PA (B) (**p < .001).

Paired sample *t*-tests revealed that ankle mean PA was significantly higher at daytime P1 (*M* = 0.63 *m/s*^*2*^, *SD* = 0.25 *m/s*^*2*^) than at daytime P2 (*M* = 0.54 *m/s*^*2*^, *SD* = 0.20 *m/s*^*2*^): *t*(46) = 2.46, *p =* .02; similarly, wrist mean PA was higher at daytime P1 (*M* = 0.62 *m/s*^*2*^, *SD* = 0.18 *m/s*^*2*^) than daytime P2 (*M* = 0.57 *m/s*^*2*^, *SD* = 0.14 *m/s*^*2*^): *t*(46) = 2.21, *p =* .03. When comparing mean PA of wrist and ankle at daytime P1, no differences were found between ankle (*M* = 0.63 *m/s*^*2*^, *SD* = 0.25 *m/s*^*2*^) and wrist (*M* = 0.62 *m/s*^*2*^, *SD* = 0.18 *m/s*^*2*^): *t*(46) = 0.45, *p =* .65. Similarly, at daytime P2, mean PA of ankle (*M* = 0.54 *m/s*^*2*^, *SD* = 0.20 *m/s*^*2*^) did not differ from wrist mean PA (*M* = 0.57 *m/s*^*2*^, *SD* = 0.14 *m/s*^*2*^): *t*(46) = 1.14, *p =* .26. (Fig 5A)

Paired *t*-tests showed that ankle PA CV was higher at daytime P1 (*M* = 0.68 *m/s*^*2*^, *SD* = 0.09 *m/s*^*2*^) than at daytime P2 (*M* = 0.65 *m/s*^*2*^, *SD* = 0.10 *m/s*^*2*^): *t*(46) = 2.82, *p =* .007, however, no differences in wrist PA CV were found between daytime P1 (*M* = 0.55 *m/s*^*2*^, *SD* = .06 *m/s*^*2*^) and daytime P2 (*M* = 0.54 *m/s*^*2*^, *SD* = 0.06 *m/s*^*2*^): *t*(46) = 1.23, *p =* .22. Comparisons of PA CV between wrist and ankle at daytime P1 revealed that PA CV of ankle (*M* = 0.68 *m/s*^*2*^, *SD* = 0.09 *m/s*^*2*^) was higher than wrist (*M* = 0.55 *m/s*^*2*^, *SD* = .06 *m/s*^*2*^): *t*(46) = 8.94, *p* < .0001. Similarly, at daytime P2, PA CV of ankle (*M* = 0.65 *m/s*^*2*^, *SD* = 0.10 *m/s*^*2*^) was higher than wrist (*M* = 0.54 *m/s*^*2*^, *SD* = .06 *m/s*^*2*^): *t*(46) = 7.37, *p* < .0001. (Fig 5B).

## Discussion

Here, we explored systematic differences of PA in terms of mean and variability between the lower and upper extremities at day and night and within-day segments for seven days continuously in healthy individuals. We show that mean activity between wrist and ankle did not differ in any period (i.e., daytime and nighttime). In contrast, intra-subject variability, as quantified by CV, differed significantly between wrist and ankle: at daytime, the ankle had higher PA variability than the wrist; whereas at nighttime, wrist PA had greater variability. For diurnal patterns, we found that mean PA was significantly reduced between the first and second halves of the daytime period. We also showed that mean and variability of both wrist and ankle PA during daytime were negatively associated with age. Although we predicted a negative association between mean PA and BMI, we only observed a negative BMI association with wrist PA variability during daytime, for which the significance was affected by an outlier (Fig 4D).

The current study was performed in a free-setting environment and participants engaged in their regular diurnal and nightly behaviors. Daily activities such as walking may require high involvement of ankle and wrist, while other behaviors such as desk or domestic activities may require higher contribution from upper limbs. Here, we show no difference of PA mean between wrist and ankle for either daytime or nighttime in healthy subjects. Thus, it appears that gross movements that engage both upper and lower extremities dominate the small magnitude movements that might differently engage the upper over the lower extremities (i.e., desk activities). This was also supported by the high correlation between mean wrist and mean ankle PA in daytime (Table 2 and Fig 3E) showing a non-significant bias for mean PA between wrist and ankle at daytime (Fig 3A). A recent study that compared activity between wrist and hip monitors over 7 days, showed that wrist activity measures reached 5-fold higher values than those of the hip, but the pattern of PA detected throughout the day was similar [42]. This could be due to the lack of back and forth movement in the hips compared to the wrists. Thus, identifying how extremities PA patterns vary according to specific activities will require a comparison between different threshold levels of sensitivity for detecting PA with accelerometers. Such studies would benefit from concurrent recordings of daily activities of both extremities.

In contrast, we found differences in intra-subject variability of PA between ankle and wrist as a function of daytime and nighttime (i.e., awake/sleep) periods (Fig 2). High ankle variability of movement during the day could be driven by the nature of diurnal activities, such as work, leisure, and commuting or domestic activities. Nocturnal intra-subject PA variability could be driven by the transitioning between REM and Non-REM sleep cycles as well as awakening episodes [56, 57]. The difference in intra-subject PA variability between the wrist and ankle as a function of time of day suggests differential diurnal control of movement in upper and lower extremities. Indeed, differential involvement of the lower and upper limbs has been reported in movement disorders at nighttime [58, 59]. Future studies are needed to evaluate the relationship between brain function and differences of PA variability between wrist and ankle at daytime and nighttime.

In addition to sleep-wake cycles, time-of-day has been recognized as a key factor for the interaction of PA with neurological and cardiovascular systems [19]. For example, in neurologic diseases, time-of-day influences walking and gait variability [60]. These observations highlight the importance of the interaction between time-of-day and PA engagement in neuro-physical health. We found significant decreases in mean PA for both ankle and wrist from P1 to P2, but only ankle CV significantly decreased from P1 to P2. These observations suggest that ankle-related measures may be more sensitive to diurnal changes of PA than wrist, which may have implications for related studies in the future.

As suggested by previous research, we found that mean PA of the lower and upper limbs decreased by age but only at daytime. After accounting for the effects of mean (i.e., CV was computed by dividing SD by mean of PA), we found that CV also significantly decreased with age during daytime. These findings suggest that age may impact both overall but also patterns of activity. Although there is evidence that movement variability increases in association with age-related neurodegenerative disorders, such as Parkinson’s disease [61], we showed a decrease in variability with age. This discrepancy may reflect the difference between the effects of aging on PA in healthy individuals versus the effects of neurodegenerative disorders on PA. In fact, time-of-day effects on ankle CV found here help contrast within-day PA characteristics of healthy versus populations with neurological disorders, whose balance control decreases and gait variability increases in the evening [60]. Furthermore, in healthy aging, a predominant contributor to PA mean and variability might relate to changes in lifestyle and motor strength whereas for Parkinson’s disease they might predominantly reflect the loss of DA neurons. Nevertheless, a limitation of this study is that the sample used was relatively small and the mean age was close to 40-years-old, which limits its generalizability to older populations. This can be improved by increasing the sample size and adding older participants.

Contrary to our hypothesis, we did not find an association between BMI and mean PA. This could also reflect the limited statistical power due to the small sample size in our study. Nevertheless, we found a significant negative association between wrist PA variability and BMI during daytime, such that the higher the BMI the lower the PA wrist variability. This association might be driven by a reduced probability of shifting between activities as a function of BMI, though this effect was not seen in ankle PA variability in our sample. Further studies are needed to determine the differential sensitivity to BMI for wrist versus ankle monitors for movements in lower versus upper extremities.

The present results suggest that quantitative assessment of mean PA only requires one accelerometer device on one body location for healthy controls. However, monitoring the variability in movement as seen in motor disorders like Parkinson’s or other age-related neurodegenerative disorders might benefit from monitoring activity in both body extremities.

## Supporting information

S1 File Physical activity and demographics raw data. Excel file containing acceleration raw data in 0.1 g units (m/s^2) for 47 participants included in all analyses. Column A indicates subject number. Columns B-E contain mean and CV data of ankle at daytime and nighttime. Columns F-I contain wrist mean and CV data for daytime and nighttime. Columns J-M contain demographics data, including gender, age, and BMI of participants.
(XLSX)
